# Deep Learning on H&E Pathology Images Predicts *KRAS* and *TP53* Mutations in Pancreatic Adenocarcinoma: A Multicenter Study

**DOI:** 10.3390/diseases14070249

**Published:** 2026-07-10

**Authors:** Dongheng Ma, Hinano Nishikubo, Tomoya Sano, Masakazu Yashiro

**Affiliations:** 1 Department of Molecular Oncology and Therapeutics, Osaka Metropolitan University Graduate School of Medicine, 1-4-3 Asahimachi, Abeno-ku, Osaka 545-8585, Japan; 2 Cancer Center for Translational Research, Osaka Metropolitan University Graduate School of Medicine, 1-4-3 Asahimachi, Abeno-ku, Osaka 545-8585, Japan

**Keywords:** pancreatic ductal adenocarcinoma, deep learning, whole-slide imaging, computational pathology, *KRAS* mutation, *TP53* mutation

## Abstract

**Background:** Pancreatic ductal adenocarcinoma (PDAC) carries a dismal prognosis, and *KRAS* and *TP53* mutational status is increasingly recognized as both prognostic and therapeutically actionable. Because next-generation sequencing has long turnaround times, high costs, and substantial tissue requirements, we aimed to develop and externally validate a deep-learning framework for inferring *KRAS* and *TP53* mutation status directly from routine hematoxylin-and-eosin (H&E) whole-slide images (WSIs) of PDAC. **Methods:** A training cohort was assembled from TCGA-PAAD (*n* = 206) and CPTAC-PAAD (*n* = 147), and an independent external validation cohort (*n* = 86) was obtained from Osaka Metropolitan University (OMU) Hospital. We benchmarked 28 model configurations per gene, comprising three pathology foundation models (CONCH-v1.5, CTransPath, and Prov-GigaPath) crossed with nine multiple-instance-learning (MIL) aggregators (ABMIL, CLAM-SB, CLAM-MB, DSMIL, TransMIL, MeanMIL, MaxMIL, AEM, and MIL-Dropout), plus a ResNet-50 + MeanMIL baseline. Performance was evaluated by patient-level five-fold cross-validation (AUC, accuracy, precision, sensitivity, and specificity) and external AUC; attention heatmaps were generated for interpretability. **Results:** For *KRAS*, CONCH-v1.5 + MeanMIL achieved the best internal AUC of 0.717 and CONCH-v1.5 + ABMIL the best external AUC of 0.705. For *TP53*, CTransPath + DSMIL achieved the best internal AUC of 0.668 and CTransPath + MeanMIL the best external AUC of 0.744. **Conclusions:** H&E-based deep learning can infer *KRAS* and *TP53* mutation status in PDAC with moderate but reproducible discrimination, supporting its potential as a low-cost upstream prescreening tool that triages candidates for confirmatory molecular sequencing and genotype-directed targeted therapy.

## 1. Introduction

Pancreatic ductal adenocarcinoma (PDAC) is the predominant form of pancreatic cancer, which is the sixth leading cause of cancer-related death worldwide [1]. PDAC is characterized by short survival, poor prognosis, an aggressive clinical course, and a high proportion of patients who are diagnosed at advanced stages [2]. Most patients present with unresectable disease and depend on chemotherapy, which carries substantial toxicity yet yields only modest survival benefits. Recently, however, therapies directed against key PDAC driver genes have achieved progress. In a phase I/II trial reported at ESMO 2025, the selective KRAS G12D inhibitor VS-7375 achieved an objective response rate of 40.7% [3]. In parallel, *TP53* mutational status has emerged as an independent prognostic and chemo-predictive biomarker in PDAC [4]. These advances are repositioning *KRAS* and *TP53* gene status from purely prognostic classifiers toward actionable biomarkers for therapeutic decision-making. KRAS is the predominant oncogenic driver of PDAC, mutated in 90% of tumors, and TP53 is the most frequently inactivated tumor-suppressor gene, altered in 70% of cases, making them the two most recurrent driver alterations in this malignancy [5]. Gene mutation profiling relies on next-generation sequencing, which is constrained by long turnaround times (3–4 weeks), high costs, and substantial tissue requirements [6]. Therefore, predictive or screening AI tools capable of inferring mutation status hold considerable clinical value.

In recent years, computational pathology has demonstrated the ability to infer key driver mutations and other molecular biomarkers directly from routine hematoxylin-and-eosin (H&E) whole-slide images (WSIs), with reported applications spanning lung, gastrointestinal, breast, colorectal, and pan-cancer settings [7,8,9,10]. Two technical advances have driven this rapid progress. First, weakly supervised multiple-instance-learning (MIL) frameworks, exemplified by ABMIL [11], CLAM [12], DSMIL [13], and TransMIL [14], represent each slide as a bag of patch-level instances. Second, pathology foundation models, including CTransPath [15], CONCH [16], and Prov-GigaPath [17], which are trained by large-scale self-supervised pretraining on millions of WSIs, serve as general-purpose tile encoders in downstream tasks and exhibit domain-specific representational capabilities acquired through pretraining on large-scale pathology data. Nevertheless, our previous benchmark study in a pan-cancer setting showed that no single foundation model dominated across all biomarker tasks [18], implying that systematic foundation model–MIL comparisons are necessary for each new biomarker target.

Despite the overall progress in computational pathology, studies dedicated to gene mutation prediction in PDAC remain limited. Here, we develop and externally validate an H&E-based deep learning framework for predicting *KRAS* and *TP53* mutation status in PDAC. Using a combined training cohort from The Cancer Genome Atlas (TCGA) and the Clinical Proteomic Tumor Analysis Consortium (CPTAC), we systematically benchmark a 3 × 9 grid comprising three foundation models (CONCH v1.5, CTransPath, and Prov-GigaPath) and nine MIL aggregators (ABMIL, CLAM-SB, CLAM-MB, DSMIL, TransMIL, MeanMIL, and MaxMIL), with ResNet-50 + MeanMIL serving as the baseline. The selected configurations are subsequently validated in an independent external cohort from Osaka Metropolitan University Hospital (OMU). To enhance interpretability, attention heatmaps are generated to highlight tissue regions associated with the predicted mutation status. This study contributes, to our knowledge, the first systematic benchmark of multiple pathology foundation models paired with multiple MIL aggregators spanning methods from 2018 to 2025, evaluated consistently across both public and in-house clinical cohorts for KRAS and TP53 mutation prediction from H&E whole-slide images alone.

## 2. Materials and Methods

### 2.1. Cohort

WSIs of pancreatic ductal adenocarcinoma were assembled from two publicly available cohorts and one institutional cohort. The TCGA-PAAD (PAAD, pancreatic adenocarcinoma) cohort contributed 206 diagnostic WSIs, together with matched somatic mutation calls and clinical annotations [19]. The CPTAC-PAAD cohort contributed 147 diagnostic WSIs with matched whole-exome sequencing-derived mutation status [20]. The two public cohorts were merged to form the training set (*n* = 353). Cases without paired *KRAS* and *TP53* mutation annotations or with non-PDAC histology on central pathology review were excluded. The external validation cohort consisted of 86 PDAC cases from OMU Hospital (Osaka, Japan), comprising both surgical resection and biopsy specimens, with *KRAS* and *TP53* status determined by clinical-grade targeted next-generation sequencing of formalin-fixed, paraffin-embedded (FFPE) tumor blocks. The use of TCGA-PAAD and CPTAC-PAAD data complied with the data-use agreements of each consortium. The OMU Hospital cohort study was conducted in accordance with the Declaration of Helsinki and was approved by the Institutional Review Board (IRB) of Osaka Metropolitan University Hospital (Approval Nos. 2022-111, 0924, and 2022-077). Written informed consent for the use of pathological specimens and clinical data for research was obtained from all participating patients or waived by the IRB for retrospective, de-identified samples in accordance with local regulations.

### 2.2. WSI Preprocessing

WSIs from the OMU cohort were digitized in-house from FFPE H&E slides using an Aperio CS2 scanner (Leica Biosystems, Nussloch, Germany) at 40× magnification (~0.25 µm/pixel) and subsequently downsampled to 20× (~0.5 µm/pixel) to align with the effective resolution of the TCGA-PAAD and CPTAC-PAAD diagnostic slides. For all WSIs, a tissue mask was first generated by creating a low-magnification thumbnail of the slide, converting the image to the HSV color space, and applying Otsu thresholding [21] to the saturation channel to separate stained tissue from the background. Pen marks, large air bubbles, out-of-focus regions, and tissue-folding artifacts were then removed. Within the tissue mask, non-overlapping tiles were extracted from each WSI. The sampling magnification and tile dimensions were adapted to the native input specification of each pathology foundation model. To mitigate stain variability across centers and scanners, all retained tiles were stain-normalized to a common reference template prior to feature extraction [22].

### 2.3. Feature Extraction and Training

Each tile was passed through a frozen pathology foundation model used as a feature extractor, using the Trident framework [23]. We compared three publicly released pathology foundation models: CTransPath, CONCH-v1.5, and Prov-GigaPath. As a baseline, we used an ImageNet-pretrained ResNet-50 [24] truncated before the final classifier as a tile encoder. All foundation models were used in their public, frozen form; no domain-specific fine-tuning of the encoder weights was performed. Slide-level prediction was framed as a weakly supervised MIL task: each WSI was represented as a bag of tile embeddings, and the model was trained to predict a binary slide-level mutation label for each gene (*KRAS*-mutant vs. *KRAS*-wild-type; *TP53*-mutant vs. *TP53*-wild-type). We benchmarked seven MIL aggregators spanning the main families used in computational pathology: ABMIL, CLAM-SB, CLAM-MB, DSMIL, TransMIL, AEM [25] and MIL-Dropout [26], together with MeanMIL and MaxMIL as simple pooling baselines. Crossing three foundation models with nine aggregators, plus the ResNet-50 + MeanMIL baseline, yielded 28 model configurations per gene. All configurations were trained separately for *KRAS* and *TP53* prediction with patient-level stratified five-fold cross-validation on the combined TCGA-PAAD + CPTAC-PAAD training cohort. Models were optimized with the AdamW [27] optimizer using a binary cross-entropy loss; class imbalance was addressed by inverse-frequency loss weighting. Performance on the internal cross-validation cohort was reported as the area under the receiver operating characteristic (ROC) curve (AUC), accuracy, precision, sensitivity, and specificity at the operating point that maximized Youden’s J statistic on the held-out training fold. Performance on the external OMU Hospital cohort was reported as the external-validation AUC (Ext-AUC). The cross-validated, top-performing configuration per gene was carried forward to the external validation analysis. Confidence intervals, where reported, were estimated by 1000 bootstrap resamples [28] of the slide-level predictions.

All experiments were implemented in Python 3.10 with PyTorch 2.8 (CUDA 12.8) on a workstation equipped with an NVIDIA RTX A6000 GPU (48 GB VRAM). Feature extraction was performed in inference mode using the Trident framework, and the resulting bag-of-features tensors were cached as HDF5 files so that downstream MIL training was fully decoupled from repeated encoder forward passes, allowing the 28-configuration sweep to be executed efficiently. Because the foundation models produce features of different dimensionalities (768 for CTransPath, 768 for CONCH-v1.5, 1536 for Prov-GigaPath, and 1024 for the ResNet-50 baseline), a learnable linear projection followed by dropout (*p* = 0.25) was applied immediately upstream of every aggregator to map all features into a common 512-dimensional hidden space so that the aggregator architectures saw identical input dimensionality across all backbones. During MIL training, each gradient step consumed one slide-level bag (batch size = 1); the initial learning rate was set to 2 × 10^−4^, the weight decay to 1 × 10^−5^, and each configuration was trained for up to 200 epochs and early-stopped when the held-out validation loss failed to improve for 20 consecutive epochs. To keep the comparison across the 28 configurations interpretable, the random seed (seed = 1) and the patient-level five-fold split were fixed across every configuration. All numerical analyses were performed with scikit-learn 1.7 and SciPy 1.15, and figures were rendered with Matplotlib 3.10.

### 2.4. Attention-Based Heatmap Interpretation

Heatmaps were produced separately for the *KRAS* and *TP53* prediction tasks, in each case using the foundation model–MIL aggregator configuration identified in the benchmark. The trained MIL aggregator was applied in inference mode to external slides, yielding a raw attention score for every tile within the tissue mask. Attention scores were min–max normalized to the range [0, 1] within each slide to compensate for between-slide differences in the absolute magnitude of attention. The normalized scores were then remapped to the spatial coordinates of their corresponding tiles, reconstructing a slide-level two-dimensional attention map.

**Figure 1:**
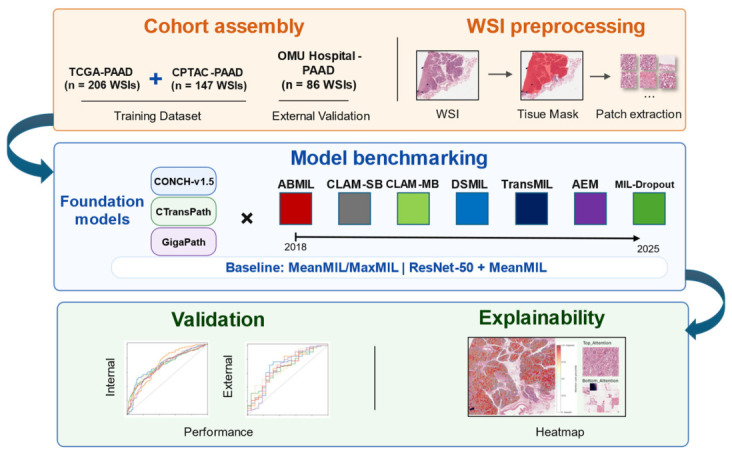
Study workflow. WSIs from TCGA-PAAD (*n* = 206) and CPTAC-PAAD (*n* = 147) were combined for training; the OMU Hospital cohort (*n* = 86) served as the external validation cohort. Pathology foundation models and MIL aggregators, together with a ResNet-50 + MeanMIL baseline, were benchmarked for KRAS and TP53 mutation prediction, followed by attention-based heatmap interpretation. Abbreviations: WSI, whole-slide image; TCGA, The Cancer Genome Atlas; CPTAC, Clinical Proteomic Tumor Analysis Consortium; PAAD, pancreatic adenocarcinoma; OMU, Osaka Metropolitan University; MIL, multiple-instance learning.

The overall study workflow is summarized in Figure 1.

## 3. Results

### 3.1. Benchmarking of Foundation Models and MIL Aggregators

We benchmarked 28 configurations for the prediction of each of the *KRAS* and *TP53* mutation statuses. Internal metrics (AUC, accuracy, precision, sensitivity, and specificity) were computed by patient-level cross-validation on the combined TCGA-PAAD + CPTAC-PAAD cohort, and external-validation AUC (Ext-AUC) was computed on the OMU Hospital cohort. Results for *KRAS* and *TP53* are summarized in Table 1.

For *TP53* mutation prediction, internal cross-validation AUCs ranged from 0.54 to 0.67 across the 28 configurations, and external-cohort AUCs ranged from 0.59 to 0.74 (Table 1). The best-performing configurations in internal cross-validation were CONCH-v1.5 + MaxMIL (AUC = 0.655) and CTransPath + DSMIL (AUC = 0.668), and the best external-cohort generalization was achieved by CTransPath + MeanMIL (Ext-AUC = 0.744; balanced accuracy = 0.67, MCC = 0.34, Brier score = 0.20) and Prov-GigaPath + DSMIL (Ext-AUC = 0.716). The ResNet-50 + MeanMIL baseline reached an internal AUC of 0.593 and an Ext-AUC of 0.648, indicating a modest but consistent improvement of the foundation-model configurations over the ImageNet-pretrained baseline.

For *KRAS* mutation prediction, internal cross-validation AUCs ranged from 0.62 to 0.72, and external-cohort AUCs from 0.54 to 0.71. The best-performing configurations in internal cross-validation were CONCH-v1.5 + MeanMIL (AUC = 0.717) and CONCH-v1.5 + DSMIL (AUC = 0.716), and the strongest external generalization was reached by CONCH-v1.5 + ABMIL (Ext-AUC = 0.705; balanced accuracy = 0.67, MCC = 0.29, Brier score = 0.20) and the ResNet-50 + MeanMIL baseline (Ext-AUC = 0.705). Several configurations exhibited a high-precision, moderate-sensitivity profile (e.g., CONCH-v1.5 + MeanMIL: precision 0.885, specificity 0.866), reflecting the strong *KRAS* class imbalance (*KRAS*-mutant prevalence in the training cohort, >80%) that biases models toward majority-class predictions.

### 3.2. ROC Curves of Top Configurations

**Figure 2:**
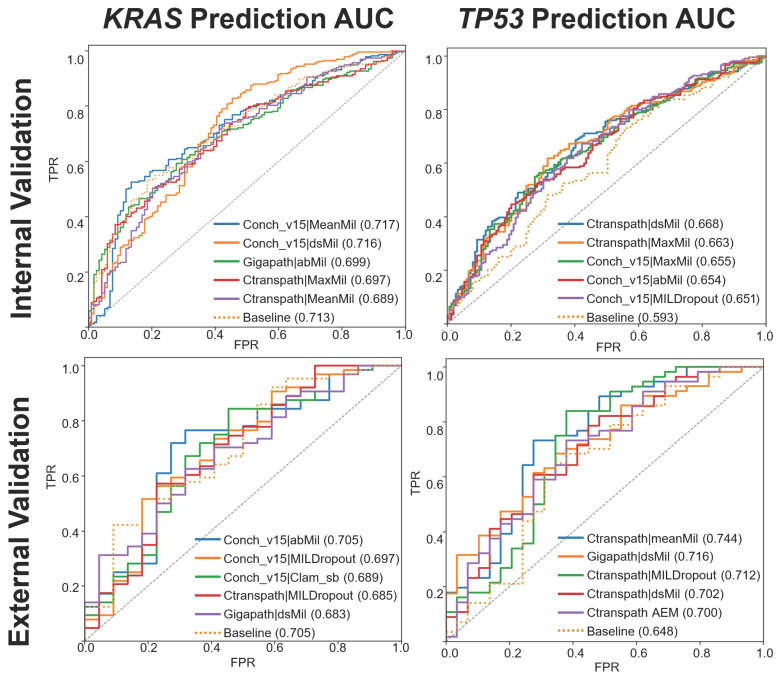
ROC curves of the top five configurations and the ResNet-50 + MeanMIL baseline for KRAS and TP53 mutation prediction in internal cross-validation (top) and external validation (bottom). Within each panel, configurations are ranked by AUC. Sample sizes: internal cross-validation cohort, *n* = 353 (KRAS-mutant 234/wild-type 119; TP53-mutant 204/wild-type 149); external validation cohort, *n* = 86 (KRAS-mutant 64/wild-type 22; TP53-mutant 57/wild-type 29). AUC (95% CI, 1,000 bootstrap resamples); Internal, KRAS: Conch_v15|MeanMil 0.717 (0.66–0.77), Conch_v15|dsMil 0.716 (0.66–0.78), Gigapath|abMil 0.699 (0.65–0.75), Ctranspath|MaxMil 0.697 (0.64–0.75), Ctranspath|MeanMil 0.689 (0.63–0.75), baseline 0.713 (0.66–0.77); Internal, TP53: Ctranspath|dsMil 0.668 (0.61–0.72), Ctranspath|MaxMil 0.663 (0.60–0.72), Conch_v15|MaxMil 0.655 (0.60–0.71), Conch_v15|abMil 0.654 (0.60–0.71), Conch_v15|MILDropout 0.651 (0.59–0.71), baseline 0.593 (0.53–0.65); External, KRAS: Conch_v15|abMil 0.705 (0.56–0.83), Conch_v15|MILDropout 0.697 (0.55–0.82), Conch_v15|Clam_sb 0.689 (0.54–0.82), Ctranspath|MILDropout 0.685 (0.54–0.82), Gigapath|dsMil 0.683 (0.55–0.81), baseline 0.705 (0.57–0.83); External, TP53: Ctranspath|MeanMil 0.744 (0.63–0.86), Gigapath|dsMil 0.716 (0.60–0.82), Ctranspath|MILDropout 0.712 (0.58–0.83), Ctranspath|dsMil 0.702 (0.58–0.82), Ctranspath|AEM 0.700 (0.59–0.81), baseline 0.648 (0.51–0.78).

Figure 2 shows the ROC curves of the top five configurations for KRAS and TP53 mutation prediction in internal cross-validation and the OMU Hospital external validation cohort. For both genes, the ROC shapes in the external cohort closely tracked the internal-cohort curves, indicating that the top-ranked models retained their discrimination on previously unseen institutional data. The narrow vertical spread of the top five ROC curves further indicates that the leading configurations were not driven by a single outlier model.

### 3.3. Attention Heatmap Interpretation

**Figure 3:**
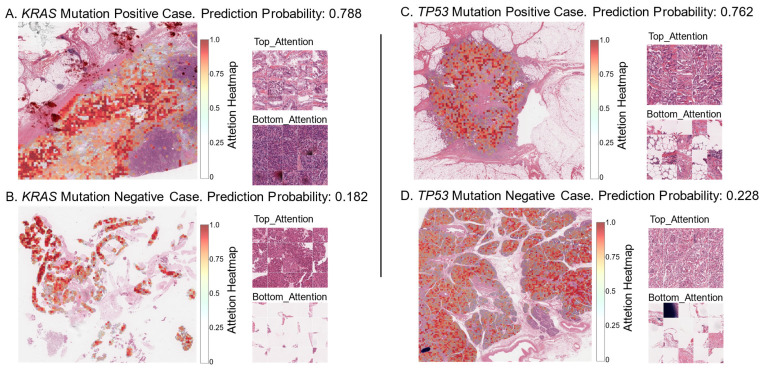
Attention heatmaps for representative external validation cases. (**A**) KRAS-mutant case (predicted probability = 0.788) and (**B**) KRAS-wild-type case (predicted probability = 0.182); (**C**) TP53-mutant case (predicted probability = 0.762) and (**D**) TP53-wild-type case (predicted probability = 0.228). For each case, the whole-slide image is overlaid with a patch-level attention heatmap (color bar: attention rank percentile), with the corresponding top- and bottom-attention patches shown on the right.

To interpret model decisions, we rendered slide-level attention heatmaps for representative *KRAS*- and *TP53*-mutation-positive and mutation-negative cases. For each case, patches with the highest and lowest attention scores (Top_Attention and Bottom_Attention patches) are shown alongside the predicted probability (Figure 3). For *KRAS*-mutant cases, high-attention patches were enriched in regions showing moderately to poorly differentiated ductal adenocarcinoma with infiltrative growth and desmoplastic stroma; for *KRAS*-wild-type cases, high-attention regions more often corresponded to better-differentiated glandular structures. For *TP53*-mutant cases, high-attention patches frequently included regions with marked nuclear pleomorphism and high-grade ductal architecture, whereas *TP53*-wild-type cases more often highlighted well-formed glands with relatively bland nuclei. Low-attention regions were consistently dominated by stroma-poor or minimally cellular tissue, indicating that the model down-weighted areas with limited diagnostic information.

## 4. Discussion

In this study, we developed and externally validated an H&E-based deep learning framework for predicting *KRAS* and *TP53* mutations in PDAC. We systematically benchmarked three pathology foundation models and nine MIL aggregators. Our results show that histology itself contains a detectable, although limited, mutation-associated signal: the best-performing configurations reached AUCs above 0.70 in both internal cross-validation and external validation. Importantly, several leading configurations also achieved sensitivity above 0.85, although the moderate overall discrimination means that these models are not yet suitable for diagnostic use and could at most serve as an upstream triage tool that flags candidate cases for confirmatory molecular sequencing. Our benchmark results showed that no single foundation model or MIL aggregator dominated across both genes and both cohorts. CONCH-v1.5 was the strongest backbone for *KRAS* prediction in the internal cohort, whereas CTransPath and Prov-GigaPath each yielded the best external AUC under different aggregator configurations. The breadth of this benchmark, together with the narrow performance spread across configurations under five-fold cross-validation and external validation, suggests that the comparison is sufficiently powered to characterize the achievable performance and that further gains will likely require richer inputs, such as multimodal integration, rather than additional aggregator variants. These findings are consistent with a prior study, which indicated that the scaling laws observed in natural-image and language-model domains do not transfer cleanly to histopathology [29]. A natural extension along this line is the integration of multimodal information. Prior work has shown that combining H&E features with preoperative cross-sectional imaging [30] or bulk transcriptomics [31] can substantially improve mutation prediction in other solid tumors. In parallel, multimodal pathology foundation models that jointly encode H&E images with paired pathology reports [32] or with transcriptomic embeddings represent an emerging route toward richer slide-level representations. Additionally, our models assigned higher attention weights to tumor regions, indicating that they captured some pathologically meaningful morphological cues; however, decoding the signal solely from a small number of top-ranked patches is unlikely to fully characterize the histopathological information distributed across an entire whole-slide image. A promising direction worth exploring is to push the analytical granularity from the patch level down to the cellular level. Constructing cell graphs would provide a finer-grained, structurally explicit representation that complements patch-based features and may further strengthen the morphological encoding of histology images [33].

A clinical implication that follows from these findings, though not fully addressed by our binary endpoint formulation, concerns how finely *KRAS* prediction can guide therapy selection. As noted in Section 1, the selective *KRAS*-G12D inhibitor VS-7375 achieved an objective response rate of 40.7% in previously treated patients with *KRAS*-G12D-mutated PDAC [3]. In this context, framing *KRAS* prediction as a binary mutant-versus-wild-type endpoint, as we and most prior studies have done, falls one informational step short of what therapy selection requires. In PDAC, G12D accounts for 39.2% of *KRAS* mutations, G12V 32.5%, and G12R 17.1% [34]. A binary positive call could not identify which specific allele a given inhibitor targets. To move closer to direct clinical impact, future work should adopt subtype-specific mutation labels rather than binary calls, allowing the model output to speak more directly to treatment decisions. A second clinical implication concerns the tissue constraints of PDAC diagnostic workup. EUS-FNB biopsy specimens are the common source of pretreatment tissue in PDAC [35], yet they often contain limited tumor cellularity. Most commercial sequencing panels require tumor content of at least approximately 20% [36], and a substantial proportion of PDAC biopsies do not reach this threshold. For patients in whom next-generation sequencing is not feasible because of insufficient tissue, an H&E-based prediction model offers a practical alternative, since a WSI-derived probability estimate can still provide a preliminary indication of mutation status to inform subsequent clinical decisions. For *TP53*, the clinical positioning of an H&E-based predictor differs from that of *KRAS* in two respects. First, our external validation showed that the best configuration for *TP53* reached an Ext-AUC of 0.744, a level of discrimination that is closer to clinical implication than what we observed for *KRAS*. Second, *TP53* mutational status serves mainly as a prognostic and chemo-predictive biomarker [4]. So, a WSI-based estimate of *TP53* status could support survival prognostication or serve as a candidate biomarker for longitudinal monitoring during treatment. Taken together, the two endpoints address distinct clinical needs, and both have a credible path to deployment within a pathology AI workflow, with the potential to reduce healthcare costs, improve diagnostic efficiency, and provide additional clinical information to support treatment decisions.

Several limitations of the present study should be acknowledged. First, although the external validation cohort is geographically independent from TCGA-PAAD and CPTAC-PAAD, its sample size is modest (*n* = 86), and larger prospective, multicenter cohorts are required. Second, the class imbalance of *KRAS* in PDAC may inflate certain evaluation metrics. Third, although attention-based interpretability is informative, it can only highlight regions whose features are associated with the model’s output and cannot establish a causal link between morphology and genotype. Despite these limitations, the present study provides a reproducible benchmark for the prediction of *KRAS* and *TP53* variants using H&E staining in PDAC across multiple foundation-model and MIL configurations. As allele-specific KRAS inhibitors such as the KRAS-G12D inhibitor VS-7375 advance into clinical use, extending these H&E-based predictors from binary mutation calls toward subtype-specific genotypes could help prioritize patients for matched targeted agents and inform the design and patient enrollment of future drug trials, representing a concrete step toward precision oncology in PDAC.

## 5. Conclusions

In this multicenter study, we developed and externally validated an H&E-based deep-learning framework for predicting *KRAS* and *TP53* mutations in PDAC. By systematically benchmarking three pathology foundation models against nine MIL aggregators on the combined TCGA-PAAD and CPTAC-PAAD training cohort and validating the leading configurations on an independent OMU Hospital cohort, we demonstrated that PDAC histology carries a measurable, although limited, signal of *KRAS* and *TP53* mutational status. The best configurations exceeded an AUC of 0.70 in external validation, and several leading configurations reached sensitivities above 0.85, indicating performance compatible with use as an upstream pre-screening tool to triage PDAC cases for confirmatory molecular sequencing and potentially reduce the diagnostic-cost burden. Future work should expand this approach to larger prospective multicenter cohorts, integrate complementary modalities such as cross-sectional imaging and bulk transcriptomics, and refine analytical granularity from the patch level toward cell-graph representations to more fully exploit the morphological information distributed across whole-slide images.

## Figures and Tables

**Table 1:** Benchmark performance across 28 configurations (3 foundation models × 9 MIL aggregators + ResNet-50 baseline) for KRAS and TP53 mutation prediction.

Gene	FM	MIL	AUC	Accuracy	Precision	Sensitivity	Specificity	Ext-AUC
*KRAS*	CONCH v1.5	ABMIL	0.673	0.615	0.818	0.538	0.765	0.705
		CLAM-SB	0.689	0.669	0.813	0.65	0.706	0.689
		CLAM-MB	0.686	0.649	0.799	0.628	0.689	0.682
		DSMIL	**0.716**	0.734	0.782	0.829	0.546	**0.675**
		TransMIL	0.676	0.66	0.779	0.679	0.622	0.577
		AEM	0.658	0.657	0.763	0.701	0.571	0.683
		MIL-Dropout	0.68	0.618	0.819	0.543	0.765	0.697
		MaxMIL	0.673	0.657	0.773	0.684	0.605	0.661
		MeanMIL	**0.717**	0.64	0.885	0.526	0.866	**0.68**
	CTransPath	ABMIL	0.678	0.626	0.811	0.568	0.739	0.683
		CLAM-SB	0.623	0.584	0.764	0.538	0.672	0.659
		CLAM-MB	0.645	0.649	0.772	0.667	0.613	0.677
		DSMIL	0.65	0.618	0.8	0.564	0.723	0.662
		TransMIL	0.66	0.618	0.783	0.585	0.681	0.576
		AEM	0.611	0.598	0.753	0.585	0.622	0.661
		MIL-Dropout	0.684	0.623	0.81	0.564	0.739	0.685
		MaxMIL	0.697	0.603	0.831	0.504	0.798	0.635
		MeanMIL	0.689	0.683	0.772	0.739	0.571	0.664
	GigaPath	ABMIL	0.699	0.637	0.808	0.594	0.723	0.624
		CLAM-SB	0.681	0.62	0.812	0.556	0.748	0.622
		CLAM-MB	0.676	0.603	0.805	0.53	0.748	0.646
		DSMIL	0.664	0.657	0.802	0.641	0.689	0.683
		TransMIL	0.636	0.62	0.758	0.628	0.605	0.535
		AEM	0.652	0.652	0.749	0.714	0.529	0.648
		MIL-Dropout	0.686	0.643	0.803	0.611	0.706	0.6
		MaxMIL	0.665	0.649	0.77	0.671	0.605	0.619
		MeanMIL	0.682	0.615	0.831	0.526	0.79	0.638
	ResNet-50 (baseline)	MeanMIL	**0.713**	0.629	0.85	0.534	0.815	**0.705**
*TP53*	CONCH v1.5	ABMIL	0.654	0.595	0.752	0.446	0.799	0.63
		CLAM-SB	0.631	0.615	0.689	0.608	0.624	0.626
		CLAM-MB	0.637	0.652	0.651	0.858	0.369	0.644
		DSMIL	0.618	0.615	0.675	0.642	0.577	0.624
		TransMIL	0.538	0.595	0.603	0.877	0.208	0.592
		AEM	0.628	0.626	0.686	0.652	0.591	0.661
		MIL-Dropout	0.65	0.632	0.685	0.672	0.577	0.651
		MaxMIL	0.655	0.626	0.728	0.564	0.711	0.67
		MeanMIL	0.629	0.635	0.658	0.765	0.456	0.65
	CTransPath	ABMIL	0.6	0.623	0.652	0.745	0.456	0.642
		CLAM-SB	0.641	0.584	0.739	0.431	0.792	0.655
		CLAM-MB	0.59	0.578	0.65	0.583	0.57	0.66
		DSMIL	0.668	0.649	0.7	0.686	0.597	0.702
		TransMIL	0.567	0.592	0.628	0.721	0.416	0.604
		AEM	0.576	0.524	0.781	0.245	0.906	0.7
		MIL-Dropout	0.601	0.575	0.673	0.515	0.658	0.712
		MaxMIL	0.663	0.646	0.728	0.618	0.685	0.682
		MeanMIL	**0.636**	0.629	0.673	0.696	0.537	**0.744**
	GigaPath	ABMIL	0.612	0.612	0.64	0.75	0.423	0.674
		CLAM-SB	0.624	0.581	0.715	0.456	0.752	0.682
		CLAM-MB	0.636	0.589	0.703	0.5	0.711	0.69
		DSMIL	0.635	0.589	0.725	0.466	0.758	0.716
		TransMIL	0.537	0.595	0.602	0.882	0.201	0.627
		AEM	0.6	0.584	0.677	0.534	0.651	0.697
		MIL-Dropout	0.634	0.575	0.741	0.407	0.805	0.661
		MaxMIL	0.596	0.606	0.67	0.627	0.577	0.672
		MeanMIL	0.602	0.567	0.689	0.456	0.718	0.656
	ResNet-50 (baseline)	MeanMIL	0.593	0.606	0.641	0.725	0.443	0.648

Internal metrics (AUC, accuracy, precision, sensitivity, and specificity) are reported for the training cohort; Ext-AUC is reported for the external validation cohort. Bold values indicate the top-performing configurations. Abbreviations: FM, foundation model; MIL, multiple-instance learning; AUC, area under the receiver operating characteristic curve; Ext-AUC, External-AUC for the external validation cohort; ABMIL, attention-based MIL; CLAM-SB, single-branch clustering-constrained attention MIL; CLAM-MB, multi-branch clustering-constrained attention MIL; DSMIL, dual-stream MIL; TransMIL, transformer-based MIL; MaxMIL, max-pooling MIL; MeanMIL, mean-pooling MIL. Values in bold indicate the best-performing model configuration for each gene.

## Data Availability

The Trident framework is available at https://github.com/mahmoodlab/TRIDENT (accessed on 20 May 2026). The data that support the findings of this study are not publicly available due to privacy and ethical restrictions.
